# Clinical safety and tolerability of *in vivo* gene editing drug ART001 for ATTR amyloidosis

**DOI:** 10.3389/fmed.2026.1783921

**Published:** 2026-04-15

**Authors:** Yasi Jiang, Lei Huang, Han Qiu, Suna Yang, Jialin Tao, Rui Chen, Yonggang Hao

**Affiliations:** 1 Department of Neurology, The Fourth Affiliated Hospital of Soochow University, Suzhou, China; 2 Clinical Research, Accuredit Therapeutics, Suzhou, China; 3 Discovery and Preclinical Research, Accuredit Therapeutics, Suzhou, China; 4 Clinical Pharmacology Research Center, Peking Union Medical College Hospital, Beijing, China; 5 State Key Laboratory of Complex Severe and Rare Diseases, Chinese Academy of Medical Sciences and Peking Union Medical College, Beijing, China

**Keywords:** ATTR, CRISPR-Cas9, gene-therapy, LNP, TTR

## Abstract

**Background:**

ATTR amyloidosis is a disease caused by abnormal deposition of TTR (transthyretin) protein in tissues. ART001 is an *in vivo* gene therapy drug using lipid nanoparticle (LNP) to deliver mRNA encoding SpCas9 (*Streptococcus pyogenes* Cas9) and a single guide RNA (sgRNA) for knocking-out of the *TTR* gene in hepatocytes and reducing serum TTR levels.

**Methods:**

In an investigator-initiated trial (IIT) of ART001 for 10 ATTR Amyloidosis patients, each patient was given one dose of ART001 which ranged from 0.05 mg/kg to 1.0 mg/kg. The aim was to evaluate ART001’s safety, side effects, PK, PD, and efficacy based on circulating TTR protein levels.

**Results:**

At 0.7 mg/kg in 3 subjects and 1 mg/kg in 3 subjects, TTR protein reductions averaged 84 and 92% at 72 weeks. No infusion-related reactions (IRRs), serious adverse events (SAEs) or serious adverse reactions (SARs) were observed.

**Conclusion:**

A single injection of ART001 achieved > 80% TTR knock-down at doses > 0.5 mg/kg and lasted for at least 72 weeks without IRRs, SARs or SAEs. ART001 has the potential to be a safe, effective and permanent therapeutic option for ATTR Amyloidosis patients. (Funded by Accuredit Therapeutics).

**Clinical trial registration:**

Trial registration: ChiCTR, ChiCTR2400081216. Registered 26^th^ Feb, 2024 - retrospectively registered, https://www.chictr.org.cn/showprojEN.html?proj=210566.

## Background

TTR protein, synthesized by hepatocytes, functions as a homotetramer in peripheral blood, transporting thyroid hormone and vitamin A. Genetic mutations or aging can destabilize the tetrameric structure, leading to TTR monomer misfolding and amyloid aggregation (1). These deposits accumulate in peripheral nerves to cause ATTR-polyneuropathy (ATTR-PN) or in the heart to induce ATTR-cardiomyopathy (ATTR-CM). Hereditary ATTR (ATTRv) is estimated to affect 10,186 individuals (range: 5,526–38,468) across 36 countries (2), with a prevalence of approximately 2,000 (range: 435–10,134) in China (3). Current treatments, including orthotopic liver transplantation and TTR-stabilizing agents like tafamidis, face limitations such as donor scarcity and transient efficacy (4, 5). Gene-silencing therapies targeting TTR mRNA, including small interfering RNAs (siRNAs) such as patisiran (6) and vutrisiran (7), and antisense oligonucleotide, such as inotersen (4, 8) and eplontersen (9), have demonstrated efficacy but require long-term, repeated dosing, underscoring the need for durable solutions.

In this context, CRISPR-Cas9–based *in vivo* gene editing has emerged as a promising therapeutic platform for ATTR amyloidosis. The CRISPR-Cas9 system comprises a Cas9 endonuclease and a single-guide RNA (sgRNA), which together form a ribonucleoprotein complex capable of inducing site-specific double-strand breaks at the *TTR* gene locus. By directly disrupting the *TTR* gene in hepatocytes, this approach offers the potential for a single-dose, durable (potentially life-long) therapy (10) *in vivo* delivery of this system typically utilizes lipid nanoparticles (LNPs), which, upon intravenous administration, are coated with apolipoprotein E and taken up selectively by hepatocytes via low-density lipoprotein receptors (11). This approach has shown promising preclinical results in multiple animal models. Mouse models carrying human TTR transgenes receiving highly modified sgRNA (G211) have demonstrated efficient TTR knockdown and reduced amyloid burden following *in vivo* genome editing (12). Similar findings were reported in cynomolgus monkeys, with durable serum TTR reduction (10). NTLA-2001 (nexiguran ziclumeran, developed by Intellia Therapeutics) was the first *in vivo* CRISPR-based therapy to enter human clinical trials for ATTR, showing ~90% sustained serum TTR reduction following a single dose in Phase I studies. NTLA-2001 has been advanced to Phase III trials, underscoring the therapeutic potential and feasibility of this strategy in clinical settings (10, 13).

ART001 is an investigational *in vivo* CRISPR-Cas9 gene-editing therapy targeting *TTR* gene developed by Accuredit Therapeutics for the treatment of ATTR amyloidosis. It employs a proprietary LNP system to deliver Cas9 mRNA and TTR-targeting sgRNA to hepatocytes, resulting in durable knockout of both wild-type and mutant TTR. In August 2023, the investigator-initiated trial (IIT) on ART001 was launched, all participants had completed at least 72 weeks of study follow-up by December 25, 2025.

Here, we report interim results from this IIT about the clinical safety, pharmacodynamics, and gene-editing efficiency of single ascending doses of ART001 in patients with hereditary ATTR.

## Methods

### Clinical study design and eligibility

**Figure 1:**
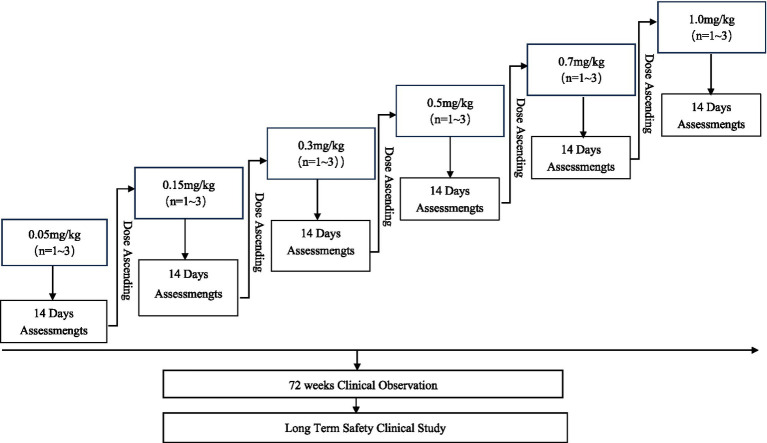
Clinical study design. Flow chart of the study design of single-center, open-label, single-arm and exploratory clinical study of ART001 in the treatment of ATTR amyloidosis. *N* = 1~3 imply that the number of the groups is flexible according to the accelerated titration scheme. All patients receive single dose in this study.

This IIT is an open-label, single-center study, including 6 dosage cohorts (Figure 1). Between August 8, 2023, and November 30, 2023, 10 patients were enrolled and completed only one ART001 administration per subject. Dosing varied from 0.05 to 1.0 mg/kg. An accelerated titration scheme is adopted to reach the therapeutic-meaningful dose quickly. Specifically, if no dose-limiting toxicity (DLT) occurs and the reduction in TTR does not reach the target level, the study may proceed directly to the next dose level on a different subject. If either condition is not met, the cohort size will be expanded to three subjects.

Inclusion criteria stipulated subjects diagnosed with ATTR-PN or ATTR-CM, aged between 18 and 80 years, and with body weights ranging from 45 to 90 kilograms. Prior use of TTR-targeting therapeutic drugs required a corresponding washout period. The doses always represent total RNA in this paper unless otherwise stated.

### Study oversight

The studies were designed by the principal investigators. Accuredit Therapeutics provided the investigational product. Data were collected by the investigators and CRC, summarised by representatives from Accuredit therapeutics, and analysed by principal investigators.

### Clinical study treatment

The planned dose groups for the study were 0.05, 0.15, 0.3, 0.5, 0.7, and 1 mg/kg. To mitigate IRRs, patients underwent pretreatment with glucocorticoids and histamine receptor type 1 and type 2 antagonists prior to the infusion of ART001. Vitamin A supplement around or over 800 IU per day was taken by all subjects during the study.

### Assessments of clinical outcomes

Patients were monitored to assess adverse events and laboratory results. Serum samples were collected at baseline and at weeks 1, 2, 4, 8, 12, 24, 36, 48, and 72 weeks for analysis of TTR protein levels using a validated immunoturbidimetric assay. No placebo group was used and the effects on TTR reduction was by comparison to the pre-dosed level of the same subject.

The Ocular Surface Disease Index (14), PN disability score (15), and FAP severity (16) was measured as cited. Immunological markers, pro-BNP, hs-cTnT and echocardiography were measured by standard clinical assays. The evaluation plan for safety outcomes is scheduled for 48 weeks post-administration of ART001. Long-term safety follow-up, as mandated by regulatory requirements, will be conducted in a separate study.

### Statistical analysis

This study was designed for descriptive analysis. Comparison between baseline and post-administration serum TTR protein levels are presented as mean percentage change.

## Results

### Patients

Eleven patients underwent initial screening for study participation. Ten patients met the inclusion criteria and were enrolled in the study (Table 1), while one patient was excluded due to positive hepatitis B serology. The enrolled cohort comprised five female and five male participants, with ages ranging from 31 to 65 years and body weights ranging from 48 to 84 kg. All enrolled patients (*n* = 10) presented sensory-motor polyneuropathy symptoms, with FAP severity ranging from 1 to 2 and Polyneuropathy Disability Scores ranging from 1 to 3a (Supplementary Table S4). Eight patients (80%) also exhibited various symptom of ATTR cardiomyopathy. Cardiac functional status was classified as New York Heart Association Class I-II heart failure in all patients with cardiac involvement.

**Table 1:** Patient demographics and baseline characteristics.

Parameter	0.05 mg/kg dose	0.15 mg/kg dose	0.3 mg/kg dose	0.5 mg/kg dose	0.7 mg/kg dose	1.0 mg/kg dose	All patients (*n* = 10)
	(*n* = 1)	(*n* = 1)	(*n* = 1)	(*n* = 1)	(*n* = 3)	(*n* = 3)	
Age, years							
Median (min, max)	64 (64)	34 (34)	55 (55)	36 (36)	46 (46, 51)	57 (36, 63)	48.5 (34, 64)
Sex, *n* (%)							
Male	1(100%)	1(100%)	0	1(100%)	0	3(100%)	6(60%)
Female	0	0	1(100%)	0	3(100%)	0	4(40%)
Weight, kg							
Median(min, max)	52 (52)	86 (86)	55 (55)	73 (73)	63 (61.5, 70)	67 (55, 82)	65 (52, 86)
Mutation status, *n* (%)							
p.T69A	0	0	0	0	1 (33%)	0	1 (10%)
p.V50L	0	0	1 (100%)	0	1 (33%)	1 (33%)	3 (30%)
p.Ala117Ser	0	0	0	0	0	1 (33%)	1 (10%)
p.Val50Met	0	0	0	0	0	1 (33%)	1 (10%)
p.Tyr134Cys	0	0	0	0	1 (33%)	0	1 (10%)
p.A56P	0	0	0	1 (100%)	0	0	1 (10%)
p.K55N	1 (100%)	1 (100%)	0	0	0	0	2 (20%)
Prior therapy, *n* (%)							
None	0	0	1 (100%)	0	0	0	1 (10%)
Tafamidis	0	1 (100%)	0	1 (100%)	2 (66%)	3 (100%)	7 (70%)
Diflunisal	1 (100%)	1 (100%)	0	1 (100%)	2 (66%)	2 (66%)	7 (70%)
Clinical scores, *n* (%)							
FAP severity							
Score 1	0	1 (100%)	1 (100%)	1 (100%)	3 (100%)	3 (100%)	9 (90%)
Score 2	1(100%)	0	0	0	0	0	1 (10%)
NYHA Functional							
Classification I	0	1 (100%)	1 (100%)	1 (100%)	3 (100%)	3 (100%)	9 (90%)
Classification II	1 (100%)	0	0	0	0	0	1 (10%)
NT-proBNP(ng/L), Median(min, max)	174 (174)	33 (33)	63 (63)	61 (61)	104 (96, 391)	106 (42, 303)	100 (33, 391)
Years since diagnosis (min, max)	2 (2)	2 (2)	2 (2)	3 (3)	0.5 (2.5, 0.5)	0.5 (0.5, 1.5)	1.75 (0.5, 3)

### Dose-escalation of ART001

The preclinical studies will be published in a separate paper (manuscript in preparation). Briefly, the off-target editing of ART001 has been assessed and no-observed-adverse-effect-level was considered to be 1 mg/kg (measured by the total RNA) from cynomolgus monkeys’ studies. In accordance with body-surface area factor of 3 and application of a safety factor of 6, the maximum recommended starting dose of ART001 for the IIT was 0.05 mg/kg.

Based on the dosed escalation principle decided in the protocol and the outcomes of the trial, eventually, one subject (4 in total) was allocated to each of the 0.05 mg/kg, 0.15 mg/kg, 0.3 mg/kg, and 0.5 mg/kg groups, and three subjects (6 in total) were assigned to each of the 0.7 mg/kg and 1.0 mg/kg groups.

### Safety and side-effect profile

**Figure 2:**
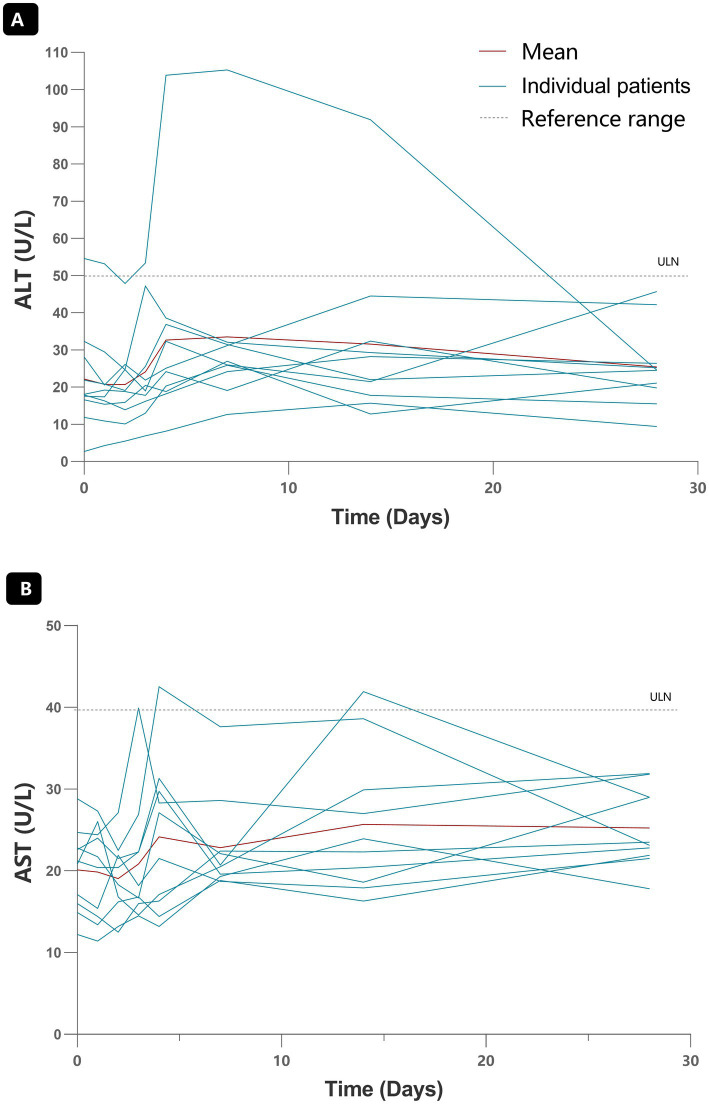
The changes in liver function tests for each patient. Each blue lines indicates one subject’s results. Red line indicates the average of all patients. Baseline is defined as the last available measurement taken prior to the start of infusion of ART001. Lower panel, changes in aspartate aminotransferase (AST); Upper panel, changes in alanine aminotransferase (ALT). ULN: Upper limit of normal.

All enrolled subjects completed a minimum of 72 weeks of follow-up. ART001 demonstrated good overall tolerability throughout the study period. As of Dec 25th, 2025, no death, SAE or Adverse Event of Special Interest (such as Grade > 1 in liver enzymes or coagulation dysfunction, IRR, or CRS) were observed following ART001 infusion. Laboratory test results revealed no clinically significant changes across all enrolled subjects. Regarding hepatic function (Figure 2), one patient in the 1.0 mg/kg group experienced transient ALT elevation to 104 IU/L on Day 4 post-infusion, representing an increase from a baseline ALT of 54 IU/L. This elevation was considered possibly related to ART001 but resolved to normal level at the Week 2 visit without intervention. Immunotoxicity assessments, including cytokines and complement examination (Supplementary Table S1) showed no concerning findings.

TEAE (Table 2) were infrequent and manageable. In the 1.0 mg/kg group, one subject with dyslipidemia during the screening period developed Grade 3 hypertriglyceridemia 3–4 days post-dosing, which normalized within 1 week following fenofibrate treatment. One subject in the 0.15 mg/kg group experienced mild hypertriglyceridemia that resolved by week 4 and was considered possibly drug-related. Two subjects experienced Grade 1 and Grade 2 weight gain, respectively, as the patients reported a significantly increased appetite after discharge.

**Table 2:** Summary of treatment-emergent adverse events.

Events	0.05 mg/kg *n* = 1	0.15 mg/kg *n* = 1	0.3 mg/kg *n* = 1	0.5 mg/kg *n* = 1	0.7 mg/kg n = 3	1.0 mg/kg n = 3
All TEAEs						
Fatigue	0	0	0	0	0	1(1/3)
Weight loss	1(1/1)	0	0	0	0	0
Weight gain	0	0	0	0	0	2(2/3)
Edema lower limb	0	0	0	0	0	1(1/3)
Dizziness	0	0	0	0	0	2(2/3)
Hypertriglyceridemia	0	1(1/1)	0	0	0	1(1/3)
ALT elevated	0	0	0	0	0	1(1/3)
Blood urea increased	0	1(1/1)	0	0	0	0
Hand pain	0	0	1(1/1)	0	0	0
Limb pain	0	0	0	0	1(1/3)	0
Hematuria	0	0	0	0	1(1/3)	0
Somnolence	0	0	0	0	1(1/3)	0
Anorexia	0	0	0	0	1(1/3)	0
Cerebral ischemia	0	0	0	0	1(1/3)	0
Drug-related TEAEs						
Fatigue	0	0	0	0	0	1(1/3)
Weight gain	0	0	0	0	0	2(2/3)
Dizziness	0	0	0	0	0	2(2/3)
Hypertriglyceridemia	0	1(1/1)	0	0	0	1(1/3)
ALT elevated	0	0	0	0	0	1(1/3)
Blood urea increased	0	1(1/1)	0	0	0	0
Grade ≥ 3 TEAEs						
Hypertriglyceridemia	0	0	0	0	0	1(1/3)
SAEs	0	0	0	0	0	0

Treatment-Emergent Adverse Events (TEAEs) are coded to the Common Terminology Criteria for Adverse Events (CTCAE) version 5.0.

Six patients from low to high dose group were followed for their Ocular Surface Disease Index (OSDI). Three patients, all in 0.7 mg/kg or 1 mg/kg groups, showed mild increase in OSDI (Supplementary Table S3) but showed no severe symptoms of dry eye or nyctalopia around Week 72.

### Reduction of TTR protein

**Figure 3:**
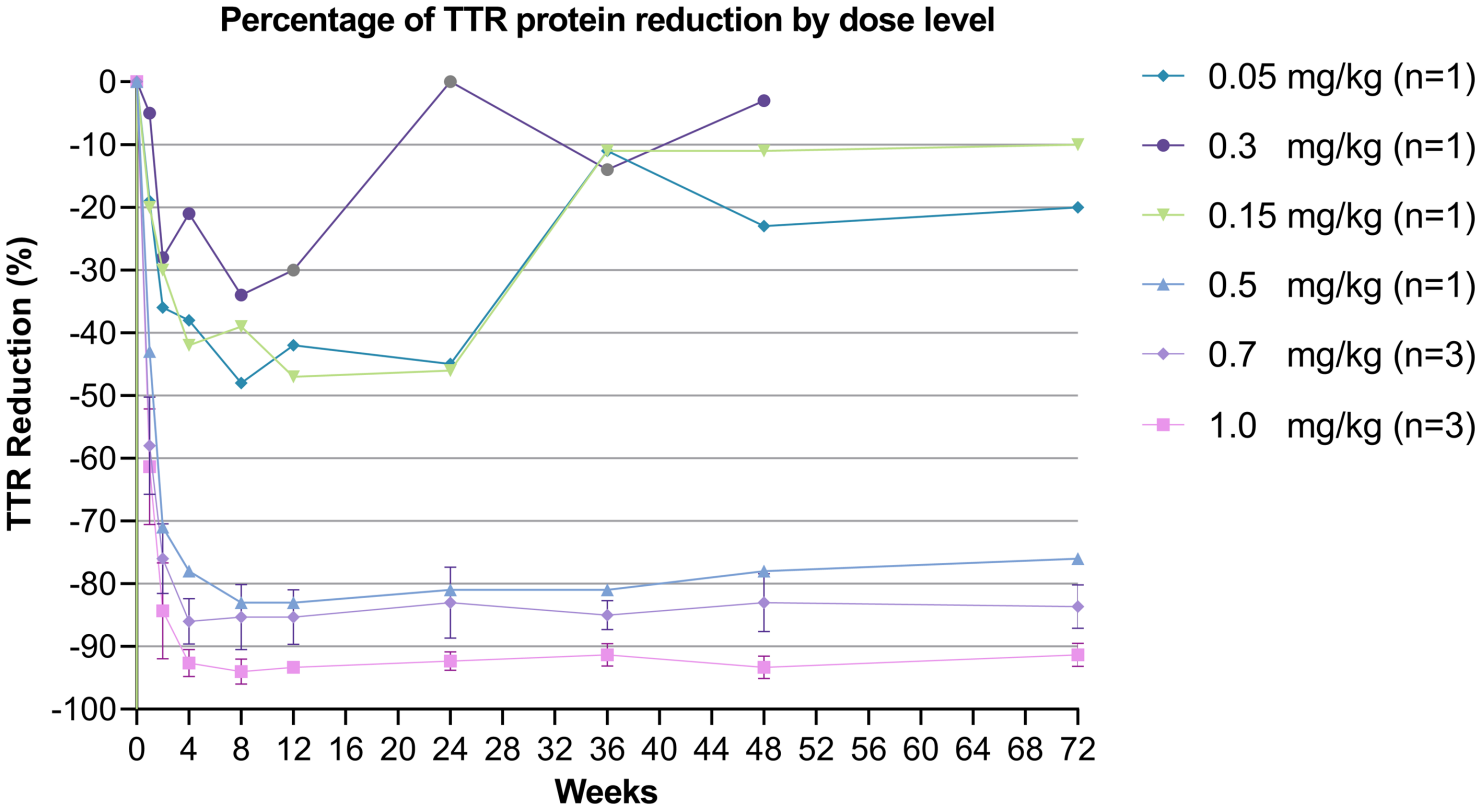
Percent change from baseline in circulating TTR protein levels over 72 weeks in human subjects. Mean serum TTR protein concentrations normalized to pre-dose level were plotted against time in weeks after receiving ART001 in 10 human subjects. Mean ± SD values were plotted (*n* = 3) for the dose of 0.7 mg/kg and 1 mg/kg. The data point for subject on 0.3 mg/kg dose at week 72 was not measured.

During the 72-week follow-up period, TTR protein reduction demonstrated dose-dependent effects within medium-to-high dosing ranges (Figure 3 for relative reduction and Supplementary Table S2 for absolute reduction at individual level). In the lower dose cohorts (0.05–0.3 mg/kg), no clear dose–response relationship was observed for TTR protein reduction, and circulating TTR levels showed fluctuation over time. In contrast, the higher dose groups (0.5, 0.7 and 1 mg/kg) exhibited robust and sustained TTR protein suppression. At 4-weeks post-treatment, serum TTR levels were reduced by 78, 86 and 92% in the 0.5, 0.7 and 1.0 mg/kg groups, respectively. This suppression was maintained throughout the study period, with TTR reductions of 76, 84 and 92% observed at 72-weeks in the corresponding dose groups.

### Efficacy measures

Though not the primary focus of this trial, some key clinical outcomes were followed and compared to pre-dosed level, when available. All six patients in 0.7 mg/kg or 1 mg/kg group’s PN disability score remained stable till week 72 (Supplementary Table S4). There is no significant changes in other outcomes, such as pro-BNP, hs-cTnT, echocardiography or FAP severity. Those observations indicate that for those 6 patients, their diseases did not progress after treatments.

## Discussion

This IIT study established the preliminary efficacy and clinical safety of ART001, a single-dose *in vivo* gene editing therapy targeting the human TTR gene, in 10 patients with ATTR amyloidosis. ART001 demonstrated robust, dose-dependent reductions in serum TTR protein levels. In the higher dose cohorts (0.5, 0.7, and 1.0 mg/kg), maximum knockdown was achieved by week 4 and sustained throughout the 72-weeks follow-up period. This observed durability following a single administration suggests a sustained therapeutic effect. The clinical safety profile characterized by predominantly mild and transient treatment-emergent adverse events without sequelae during the 72-week follow-up period supports ART001’s potential as a well-tolerated, long-acting gene editing therapy with the potential to transform the treatment paradigm for ATTR amyloidosis. To our knowledge, this study also represents the first clinical evaluation of *in vivo* LNP-delivered gene editing therapeutics in an Asian population.

The magnitude and durability of TTR protein reduction are widely employed pharmacodynamic markers in the development of ATTR-targeted therapies. Of note, in the dose group of 0.7 and 1.0 mg/kg, mean TTR reductions of 86 and 92% were observed at Week 4, respectively, and maintained as 84 and 92% at Week 72. Most TTR-directed treatments reduce circulating TTR levels, whereas tafamidis functions primarily as a TTR stabilizer and is associated with increased circulating TTR concentrations. Previously approved therapies, including patisiran, vutrisiran, inotersen and eplontersen have demonstrated 70–86% TTR knockdown with regular and repeated dosing (every several weeks or months) (5–7). *In vivo* gene editing approaches, such as NTLA-2001 and ART001, have shown sustained TTR reductions following a single infusion in clinical studies. Longer-term follow-up and larger studies will be necessary to further characterize the durability and clinical implications of these pharmacodynamic effects.

The clinical safety profile of LNP-delivered *in vivo* gene editing therapies may be primarily determined by LNP compositions, formulation characteristics and dose selection. No IRRs were observed with ART001 across all dose levels in this study. Furthermore, liver enzyme elevations with ART001 were limited to Grade 1 in a single patient in the 1.0 mg/kg group, who had elevated ALT baseline prior to administration. No delayed-onset or progressive liver enzyme elevations were observed following ART001 administration through completion of 72-week follow-up. In previously reported studies of other LNP-based gene editing therapies, infusion-related reactions and liver enzyme elevations have been described, with varying incidence and severity across dose levels. For example, clinical data from NTLA-2001 reported elevations in transaminases in a subset of treated subjects, including delayed-onset cases at higher dose levels. Isolated cases of high-grade liver enzyme elevations have also been reported in this therapeutic class. Continued monitoring of hepatic safety remains important for all LNP-delivered gene editing approaches. According to the 24-month report of ATTR-PN phase I trial of NTLA-2001, 6 out of 36 subjects receiving NTLA-2001 (approximately 17%) reported AST elevation (17). Notably, in the 1.0 mg/kg and fixed-dose equivalent (80 mg) cohorts, 6 out of 14 subjects (approximately 43%) experienced delayed-onset elevations of AST and/or ALT. The ATTR-CM clinical trial of NTLA-2001 also reported a death case following a delayed liver enzyme elevation (13, 17–19). Verve-101, another LNP-delivered gene editing drug (20), also showed increased liver transaminases in both preclinical NHP study and HEART-1 clinical trial (≥Grade 3) at 0.6 mg/kg (21), and they are currently developing another LNP-based gene editing product Verve-102 with an improved LNP delivery system. In the trial of LNP-based *in vivo* gene editing drug YOLT-101, two of three subjects in 0.6 mg/kg group shown > 3X ULN ALT and AST elevation (22).

In the present study, hypertriglyceridemia observed in several subjects during this study period could be related to the study drug; however, a potential association with comorbidities and corticosteroid premedication should also be considered (23). Further investigation in larger populations will be needed to clarify this association. Approved siRNA-based therapies also have shown safety signals. Patisiran (administered intravenously every 3 weeks) has been associated with infusion-related reactions, requiring premedication in a notable proportion of patients (6). Vutrisiran, while offering convenience of subcutaneous administration, requires ongoing dosing and has been associated with high frequency of upper respiratory tract infection and arthralgia (24). Antisense oligonucleotides such as inotersen have been associated with thrombocytopenia and glomerulonephritis (25). The differing administration schedules and safety profiles across therapeutic modalities highlight the importance of individualized risk–benefit assessment in ATTR management.

The gene editing platform underlying ART001 is based on a modular CRISPR-Cas9 system delivered via LNP. In principle, this platform allows reprogramming of guide RNAs to target alternative genes of interest. Early clinical investigation of ART001 has demonstrated precise and efficient editing in humans with a favorable clinical safety profile and feasibility of achieving sustained gene editing. Current ongoing and planned clinical programs will further evaluate the potential of this CRISPR-based platform. The 72-week results from this study support the continued clinical investigation of single-dose *in vivo* genome editing strategies for ATTR amyloidosis.

TTR is a carrier of retinol binding protein, which bound to vitamin A and knock-down of TTR in human by siRNA reduces serum vitamin A level (26). In our trial, when supplemented with Vitamin A, no severe symptoms of nyctalopia were observed. These observations suggest that TTR reduction, in the presence of Vitamin A supplement, does not necessarily lead to severe dry eye symptoms or nyctalopia, which are typically associated with vitamin A deficiency (27). This is consistent with the report that retinyl esters can also be transported to target cells independently of TTR (28). Subjects adhering to the standard vitamin A supplementation may not experience severe deficiency.

Exploratory clinical endpoints relevant to ATTR-PN were assessed during follow-up. No statistically significant improvement or worsening was observed over 72 weeks. Although ATTR-PN patients may have concomitant cardiac involvement, enrolled subjects in this study had minimal cardiac manifestations at baseline, and no evidence of cardiac progression was detected. Given the limited sample size and exploratory nature of these assessments, the impact of ART001 on disease progression or symptom improvement remains to be further evaluated in larger studies. In previously reported clinical trials of ATTR-PN therapies, including RNA-targeting agents such as patisiran, vutrisiran, inotersen, and eplontersen, neurological endpoints have demonstrated stabilization or modest improvement over study periods ranging from 18 to 24 months (6, 7, 25, 29). In some studies, marginal clinical benefits compared to placebo was detectable within the first 9–15 months (7, 25, 29); however, the magnitude and timing of such benefit varied across trials and patient populations. These findings suggest that while pharmacodynamic reduction of circulating TTR can occur rapidly, measurable clinical improvement may require longer follow-up and adequately powered comparative studies.

Several limitations should be acknowledged. Firstly, the small sample size of ten participants distributed across six dose cohorts limited statistical analysis generalizability or dose–response modelling. Secondly, while the durability of TTR knockdown has been demonstrated through 72 weeks, the follow-up duration remains relatively short considering the potentially life-long nature of ATTR amyloidosis, and long-term clinical benefits and safety beyond this timeframe remain unknown. Thirdly, the trial is retrospectively registered, which was an administrative oversight on our part. Lastly, this open-label, non-randomized IIT study does not provide placebo-controlled efficacy or definitive clinical outcome measures. Building upon these promising results, in 2024, ART001 obtained IND approval in both China NMPA and the United States FDA. It is the first LNP-delivered *in vivo* gene editing drug to clear IND in both countries. The Phase I/IIa trials for ATTR-PN and ATTR-CM are currently underway to further evaluate the efficacy, durability, and safety of ART001 in larger patient population. A pivotal Phase 3 study supporting regulatory approval has also been scheduled. Parallel efforts are exploring correlations between serum TTR reduction and clinical outcomes, including cardiac function, neurologic status, and patient-reported quality of life. These analyses will help establish robust biomarker-based therapeutic benefit predictors and extend the applicability of ART001 to broader patient populations while refining its role in disease management.

## Conclusion

In summary, ART001 represents a transformative single-dose gene editing therapy delivering efficient, durable, and clinically meaningful serum TTR reductions with a favorable clinical safety profile. Compared to existing TTR-lowering agents, ART001 offers differentiated advantages in safety, efficacy and convenience as a single-dose administration. These findings highlight the transformative potential of ART001 as a disease-modifying therapy for ATTR and support its advancement as a next-generation treatment capable of redefining the therapeutic paradigm in this field.

## Data Availability

The raw data supporting the conclusions of this article will be made available by the authors, without undue reservation.
