# Supplementary material for: Clinical safety and tolerability of in vivo gene editing drug ART001 for ATTR amyloidosis
Source: Front Med (Lausanne). 2026 Apr 15;13:1783921. doi: 10.3389/fmed.2026.1783921 (PMC13124466; doi:10.3389/fmed.2026.1783921)
Supplement: Supplementary file 1 [file Table_1.DOCX]

***Supplemental information of Clinical Safety and Tolerability of in vivo Gene Editing Drug ART001 for ATTR Amyloidosis***

Yasi Jiang,

Department of Neurology, The Fourth Affiliated Hospital of Soochow University

Lei Huang, Accuredit Therapeutics

Han Qiu, Accuredit Therapeutics

Suna Yang, Accuredit Therapeutics

Jialin Tao, Accuredit Therapeutics

Rui Chen, Clinical Pharmacology Research Center, Peking Union Medical College Hospital, Beijing, China; State Key Laboratory of Complex Severe and Rare Diseases, Chinese Academy of Medical Sciences & Peking Union Medical College, Beijing, China

Yonggang Hao

Department of Neurology, The Fourth Affiliated Hospital of Soochow University

[hyg3625@126.com](mailto:hyg3625@126.com)；

#### **Clinicals**

Table S 1 Immunotoxicity assessments of all the subjects within 4 weeks after dosing

| **dose group**  **mg/kg** | **Subjects ID** | **Indicators** | **ULN** | **LLN** | **D1 pre-dose** | **D1 3h** | **D2** | **D4** | **W1** | **W2** | **W4** |
| --- | --- | --- | --- | --- | --- | --- | --- | --- | --- | --- | --- |
| 0.05 | 01-002 | C3 | 0.9 | 1.8 | 0.75 | 0.66 | 0.68 | 0.63 | 0.73 | 0.69 | 0.66 |
|  |  | C4 | 0.1 | 0.4 | 0.51 | 0.23 | 0.22 | 0.2 | 0.22 | 0.2 | 0.2 |
|  |  | IL-2 | 0 | 11.4 | ＜2.5 | ＜2.5 | ＜2.5 | ＜2.5 | ＜2.5 | ＜2.5 | ＜2.5 |
|  |  | IL-4 | 0 | 12.9 | ＜2.5 | ＜2.5 | ＜2.5 | ＜2.5 | ＜2.5 | ＜2.5 | ＜2.5 |
|  |  | IL-6 | 0 | 20 | ＜2.5 | ＜2.5 | ＜2.5 | 2.814 | ＜2.5 | 2.619 | ＜2.5 |
|  |  | IL-10 | 0 | 5.9 | ＜2.5 | ＜2.5 | ＜2.5 | ＜2.5 | ＜2.5 | ＜2.5 | ＜2.5 |
|  |  | TNF-α | 0 | 5.5 | ＜2.5 | ＜2.5 | ＜2.5 | ＜2.5 | ＜2.5 | ＜2.5 | ＜2.5 |
|  |  | IFN-γ | 0 | 17.3 | ＜2.5 | ＜2.5 | ＜2.5 | ＜2.5 | ＜2.5 | ＜2.5 | ＜2.5 |
| 0.15 | 01-001 | C3 | 0.9 | 1.8 | 1.28 | 1.21 | 1.14 | 1.01 | 1.13 | 1.11 | 1.1 |
|  |  | C4 | 0.1 | 0.4 | 0.33 | 0.31 | 0.27 | 0.23 | 0.28 | 0.29 | 0.29 |
|  |  | IL-2 | 0 | 11.4 | ＜2.5 | ＜2.5 | ＜2.5 | ＜2.5 | ＜2.5 | ＜2.5 | ＜2.5 |
|  |  | IL-4 | 0 | 12.9 | ＜2.5 | ＜2.5 | ＜2.5 | ＜2.5 | ＜2.5 | ＜2.5 | ＜2.5 |
|  |  | IL-6 | 0 | 20 | ＜2.5 | ＜2.5 | ＜2.5 | ＜2.5 | ＜2.5 | ＜2.5 | 3.395 |
|  |  | IL-10 | 0 | 5.9 | ＜2.5 | ＜2.5 | ＜2.5 | ＜2.5 | ＜2.5 | ＜2.5 | 6.253 |
|  |  | TNF-α | 0 | 5.5 | ＜2.5 | ＜2.5 | ＜2.5 | ＜2.5 | ＜2.5 | ＜2.5 | ＜2.5 |
|  |  | IFN-γ | 0 | 17.3 | ＜2.5 | ＜2.5 | ＜2.5 | ＜2.5 | ＜2.5 | ＜2.5 | ＜2.5 |
| 0.3 | 01-007 | C3 | 0.9 | 1.8 | 1.07 | 1.07 | 1.01 | 1.04 | 1.05 | 1.1 | 1.18 |
|  |  | C4 | 0.1 | 0.4 | 0.31 | 0.31 | 0.28 | 0.27 | 0.28 | 0.35 | 0.4 |
|  |  | IL-2 | 0 | 11.4 | ＜2.5 | ＜2.5 | ＜2.5 | ＜2.5 | ＜2.5 | ＜2.5 | ＜2.5 |
|  |  | IL-4 | 0 | 12.9 | ＜2.5 | ＜2.5 | ＜2.5 | ＜2.5 | ＜2.5 | ＜2.5 | ＜2.5 |
|  |  | IL-6 | 0 | 20 | ＜2.5 | ＜2.5 | ＜2.5 | ＜2.5 | ＜2.5 | ＜2.5 | 2.749 |
|  |  | IL-10 | 0 | 5.9 | ＜2.5 | 6.809 | ＜2.5 | ＜2.5 | 2.698 | ＜2.5 | ＜2.5 |
|  |  | TNF-α | 0 | 5.5 | ＜2.5 | ＜2.5 | ＜2.5 | ＜2.5 | ＜2.5 | ＜2.5 | ＜2.5 |
|  |  | IFN-γ | 0 | 17.3 | ＜2.5 | ＜2.5 | ＜2.5 | ＜2.5 | ＜2.5 | 3.289 | ＜2.5 |
| 0.5 | 01-006 | C3 | 0.9 | 1.8 | 0.9 | 0.74 | 0.81 | 0.85 | 0.87 | 0.84 | 0.87 |
|  |  | C4 | 0.1 | 0.4 | 0.3 | 0.25 | 0.24 | 0.25 | 0.25 | 0.29 | 0.26 |
|  |  | IL-2 | 0 | 11.4 | ＜2.5 | ＜2.5 | ＜2.5 | ＜2.5 | ＜2.5 | ＜2.5 | ＜2.5 |
|  |  | IL-4 | 0 | 12.9 | ＜2.5 | ＜2.5 | ＜2.5 | ＜2.5 | ＜2.5 | ＜2.5 | ＜2.5 |
|  |  | IL-6 | 0 | 20 | ＜2.5 | ＜2.5 | ＜2.5 | ＜2.5 | ＜2.5 | ＜2.5 | ＜2.5 |
|  |  | IL-10 | 0 | 5.9 | ＜2.5 | 7.592 | ＜2.5 | ＜2.5 | ＜2.5 | ＜2.5 | ＜2.5 |
|  |  | TNF-α | 0 | 5.5 | ＜2.5 | ＜2.5 | ＜2.5 | ＜2.5 | ＜2.5 | ＜2.5 | ＜2.5 |
|  |  | IFN-γ | 0 | 17.3 | ＜2.5 | ＜2.5 | ＜2.5 | ＜2.5 | ＜2.5 | ＜2.5 | ＜2.5 |
| 0.7 | 01-004 | C3 | 0.9 | 1.8 | 0.67 | 0.71 | 0.66 | 0.73 | 0.71 | 0.72 | 0.72 |
|  |  | C4 | 0.1 | 0.4 | 0.14 | 0.15 | 0.13 | 0.15 | 0.16 | 0.16 | 0.13 |
|  |  | IL-2 | 0 | 11.4 | ＜2.5 | ＜2.5 | ＜2.5 | ＜2.5 | 2.971 | 5.487 | ＜2.5 |
|  |  | IL-4 | 0 | 12.9 | ＜2.5 | ＜2.5 | ＜2.5 | ＜2.5 | ＜2.5 | ＜2.5 | ＜2.5 |
|  |  | IL-6 | 0 | 20 | ＜2.5 | 2.571 | ＜2.5 | ＜2.5 | 7.801 | ＜2.5 | 2.63 |
|  |  | IL-10 | 0 | 5.9 | 9.426 | ＜2.5 | ＜2.5 | ＜2.5 | 2.693 | 5.597 | ＜2.5 |
|  |  | TNF-α | 0 | 5.5 | ＜2.5 | ＜2.5 | 2.85 | ＜2.5 | ＜2.5 | ＜2.5 | ＜2.5 |
|  |  | IFN-γ | 0 | 17.3 | 2.9 | 3.671 | ＜2.5 | ＜2.5 | 2.743 | 3.95 | ＜2.5 |
|  | 01-012 | C3 | 0.9 | 1.8 | 1.49 | 1.39 | 1.38 | 1.2 | 1.34 | 1.72 | 1.58 |
|  |  | C4 | 0.1 | 0.4 | 0.59 | 0.56 | 0.53 | 0.49 | 0.57 | 0.76 | 0.68 |
|  |  | IL-2 | 0 | 11.4 | ＜2.5 | ＜2.5 | ＜2.5 | ＜2.5 | ＜2.5 | ＜2.5 | ＜2.5 |
|  |  | IL-4 | 0 | 12.9 | 3.614 | 2.503 | ＜2.5 | ＜2.5 | ＜2.5 | ＜2.5 | ＜2.5 |
|  |  | IL-6 | 0 | 20 | 9.868 | ＜2.5 | ＜2.5 | ＜2.5 | 7.083 | 7.08 | 15.102 |
|  |  | IL-10 | 0 | 5.9 | 5.02 | 8.941 | ＜2.5 | 3.288 | ＜2.5 | 2.837 | 3.07 |
|  |  | TNF-α | 0 | 5.5 | 5.035 | ＜2.5 | 3.808 | ＜2.5 | ＜2.5 | ＜2.5 | ＜2.5 |
|  |  | IFN-γ | 0 | 17.3 | 6.746 | ＜2.5 | ＜2.5 | ＜2.5 | ＜2.5 | ＜2.5 | ＜2.5 |
|  | 01-013 | C3 | 0.9 | 1.8 | 0.9 | 0.88 | / | 0.89 | 0.84 | 1.08 | 0.99 |
|  |  | C4 | 0.1 | 0.4 | 0.31 | 0.28 | / | 0.33 | 0.31 | 0.42 | 0.35 |
|  |  | IL-2 | 0 | 11.4 | ＜2.5 | ＜2.5 | ＜2.5 | ＜2.5 | ＜2.5 | / | ＜2.5 |
|  |  | IL-4 | 0 | 12.9 | ＜2.5 | ＜2.5 | ＜2.5 | ＜2.5 | ＜2.5 | / | ＜2.5 |
|  |  | IL-6 | 0 | 20 | ＜2.5 | ＜2.5 | ＜2.5 | ＜2.5 | 3.396 | / | ＜2.5 |
|  |  | IL-10 | 0 | 5.9 | 2.993 | 6.95 | ＜2.5 | ＜2.5 | ＜2.5 | / | 4.354 |
|  |  | TNF-α | 0 | 5.5 | ＜2.5 | ＜2.5 | ＜2.5 | ＜2.5 | 3.542 | / | ＜2.5 |
|  |  | IFN-γ | 0 | 17.3 | ＜2.5 | ＜2.5 | ＜2.5 | ＜2.5 | ＜2.5 | / | ＜2.5 |
| 1.0 | 01-009 | C3 | 0.9 | 1.8 | 0.84 | 0.82 | 0.77 | 0.74 | 0.9 | 0.92 | 0.97 |
|  |  | C4 | 0.1 | 0.4 | 0.21 | 0.22 | 0.2 | 0.19 | 0.21 | 0.23 | 0.3 |
|  |  | IL-2 | 0 | 11.4 | ＜2.5 | ＜2.5 | ＜2.5 | ＜2.5 | ＜2.5 | ＜2.5 | ＜2.5 |
|  |  | IL-4 | 0 | 12.9 | ＜2.5 | ＜2.5 | ＜2.5 | ＜2.5 | ＜2.5 | ＜2.5 | ＜2.5 |
|  |  | IL-6 | 0 | 20 | ＜2.5 | ＜2.5 | ＜2.5 | ＜2.5 | ＜2.5 | 4.288 | ＜2.5 |
|  |  | IL-10 | 0 | 5.9 | ＜2.5 | 3.981 | ＜2.5 | ＜2.5 | ＜2.5 | 6.036 | 4.027 |
|  |  | TNF-α | 0 | 5.5 | ＜2.5 | ＜2.5 | ＜2.5 | ＜2.5 | ＜2.5 | ＜2.5 | ＜2.5 |
|  |  | IFN-γ | 0 | 17.3 | ＜2.5 | ＜2.5 | ＜2.5 | ＜2.5 | ＜2.5 | ＜2.5 | ＜2.5 |
|  | 01-008 | C3 | 0.9 | 1.8 | 1.06 | 1.04 | 1.03 | 0.94 | 1.02 | 1 | 1.12 |
|  |  | C4 | 0.1 | 0.4 | 0.34 | 0.35 | 0.32 | 0.32 | 0.27 | 0.34 | 0.46 |
|  |  | IL-2 | 0 | 11.4 | ＜2.5 | ＜2.5 | ＜2.5 | ＜2.5 | ＜2.5 | ＜2.5 | ＜2.5 |
|  |  | IL-4 | 0 | 12.9 | ＜2.5 | ＜2.5 | ＜2.5 | ＜2.5 | ＜2.5 | ＜2.5 | ＜2.5 |
|  |  | IL-6 | 0 | 20 | ＜2.5 | ＜2.5 | ＜2.5 | 9.601 | 10.194 | ＜2.5 | 2.473 |
|  |  | IL-10 | 0 | 5.9 | ＜2.5 | ＜2.5 | ＜2.5 | 3.955 | 3.408 | ＜2.5 | ＜2.5 |
|  |  | TNF-α | 0 | 5.5 | ＜2.5 | ＜2.5 | ＜2.5 | ＜2.5 | ＜2.5 | ＜2.5 | ＜2.5 |
|  |  | IFN-γ | 0 | 17.3 | ＜2.5 | ＜2.5 | ＜2.5 | ＜2.5 | ＜2.5 | ＜2.5 | ＜2.5 |
|  | 01-011 | C3 | 0.9 | 1.8 | 1.13 | 1.26 | 1.16 | 1.01 | 1.21 | 1.36 | 1.32 |
|  |  | C4 | 0.1 | 0.4 | 0.42 | 0.45 | 0.39 | 0.3 | 0.57 | 0.53 | 0.55 |
|  |  | IL-2 | 0 | 11.4 | ＜2.5 | ＜2.5 | ＜2.5 | ＜2.5 | ＜2.5 | ＜2.5 | ＜2.5 |
|  |  | IL-4 | 0 | 12.9 | 4.173 | ＜2.5 | ＜2.5 | ＜2.5 | ＜2.5 | ＜2.5 | ＜2.5 |
|  |  | IL-6 | 0 | 20 | ＜2.5 | 5.235 | ＜2.5 | 3.536 | 4.811 | ＜2.5 | ＜2.5 |
|  |  | IL-10 | 0 | 5.9 | 5.509 | ＜2.5 | 3.95 | 5.9 | 3.58 | 3.444 | ＜2.5 |
|  |  | TNF-α | 0 | 5.5 | ＜2.5 | ＜2.5 | ＜2.5 | ＜2.5 | ＜2.5 | ＜2.5 | ＜2.5 |
|  |  | IFN-γ | 0 | 17.3 | ＜2.5 | ＜2.5 | ＜2.5 | ＜2.5 | ＜2.5 | ＜2.5 | ＜2.5 |

Table S 2 **Individual and Mean (SE) Serum TTR Protein Level (ug/mL)**

| **Dose group** | **Patient ID** | **Baseline*** | **W1** | **W2** | **W4** | **W8** | **W12** | **W24** | **W36** | **W48** | **W72** |
| --- | --- | --- | --- | --- | --- | --- | --- | --- | --- | --- | --- |
| **0.05 mg/kg** | 01-002 | 172 | 139 | 109 | 107 | 89 | 100 | 95 | 153 | 125 | 205 |
| **0.15 mg/kg** | 01-001 | 290 | 231 | 204 | 169 | 177 | 154 | 158 | 151 | 235 | 224 |
| **0.30 mg/kg** | 01-007 | 220 | 210 | 159 | 173 | 145 | 155 | 220 | 189 | 216 | ND |
| **0.50 mg/kg** | 01-006 | 281 | 160 | 81 | 62 | 48 | 48 | 54 | 54 | 52 | 54 |
| **0.70 mg/kg** | 01-004 | 140 | 43 | 24 | 13 | 9 | 8 | 9 | 16 | 9 | 9 |
|  | 01-012 | 210 | 79 | 42 | 25 | 30 | 41 | 41 | 32 | 36 | 36 |
|  | 01-013 | 170 | 97 | 60 | 36 | 40 | 30 | 42 | 32 | 43 | 37 |
|  | **Mean** | 173 | 73 | 42 | 25 | 26 | 26 | 31 | 27 | 29 | 27 |
|  | **SE** | 35 | 27 | 18 | 12 | 16 | 17 | 19 | 9 | 18 | 16 |
| **1.0 mg/kg** | 01-009 | 237 | 90 | 30 | 22 | 18 | 18 | 20 | 28 | 23 | 19 |
|  | 01-008 | 299.5 | 165 | 90 | 31 | 25 | 22 | 30 | 23 | 18 | 32 |
|  | 01-011 | 302.5 | 71 | 13 | 10 | 7 | 16 | 16 | 17 | 11 | 13 |
|  | **Mean** | 280 | 109 | 44 | 21 | 17 | 19 | 22 | 23 | 17 | 23 |
|  | **SE** | 37 | 50 | 40 | 11 | 9 | 3 | 7 | 6 | 6 | 10 |

Note:* Baseline TTR was the mean values of TTR measured at 3 times before administration, and the detection method of TTR was immunoturbidimetry; W = Week. ND = Not Determined.

Table S 3 **Individual OSDI score from 6 patients.**

| **Subject ID** | **Dose (mg/kg)** | **Time Point** | **score** |
| --- | --- | --- | --- |
| 01-001 | 0.15 | Baseline * | 0 |
|  |  | W72 | 0 |
| 01-002 | 0.05 | Baseline * | 10.42 |
|  |  | W72 | 10.42 |
| 01-004 | 0.70 | Baseline * | 50 |
|  |  | W72 | 75 |
| 01-006 | 0.50 | Baseline * | 6.25 |
|  |  | W72 | 6.25 |
| 01-011 | 1.0 | Baseline * | 0 |
|  |  | W72 | 37.5 |
| 01-012 | 0.70 | Baseline * | 8.3 |
|  |  | W72 | 25 |

*: Retrospectively evaluated

Table S 4 **Individual PN disability score at baseline, W24, W48 and W72.**

| **Subject ID** | **Dose group** | **baseline** | **W24** | **W48** | **W72** |
| --- | --- | --- | --- | --- | --- |
| 01-002 | 0.05mg/kg | 3a | 3a | 3a | 4 |
| 01-001 | 0.15mg/kg | 1 | 1 | 1 | 1 |
| 01-007 | 0.3mg/kg | 1 | 2 | 2 | ND |
| 01-006 | 0.5mg/kg | 1 | 1 | 1 | 1 |
| 01-004 | 0.7mg/kg | 2 | 2 | ND | 1 |
| 01-012 | 0.7mg/kg | 3a | 3a | 3a | 3a |
| 01-013 | 0.7mg/kg | 2 | 2 | 2 | 2 |
| 01-008 | 1.0 mg/kg | 2 | 2 | 2 | ND |
| 01-009 | 1.0 mg/kg | 1 |  | 2 | 2 |
| 01-011 | 1.0 mg/kg | 1 | 2 | ND | 2 |

ND: Not Determined
